# Supplementary material for: Patterns of Healthcare Utilization Leading to Diagnosis of Young-Onset Colorectal Cancer (yCRC): Population-Based Case-Control Study
Source: Cancers (Basel). 2022 Aug 31;14(17):4263. doi: 10.3390/cancers14174263 (PMC9454837; doi:10.3390/cancers14174263)
Supplement: Supplementary file 1 [file cancers-14-04263-s001.zip › cancers-1846719-supplementary.pdf]

Supplementary Table S1. Healthcare utilization among yCRC and aCRC controls during prediagnosis period (years -5 to -1) and year of diagnosis (year 1).

	Prediagnosis Period					Year of Diagnosis
	**Year -5**	**Year -4**	**Year -3**	**Year -2**	**Year -1**	**Year 0**
Outpatient visits						
yCRC (median)	3	3	4	4	8	25
aCRC (median)	6	7	7	8	12	26
yCRC (mean)	5.80 ± 8.06	5.85 ± 8.29	6.08 ± 9.01	6.23 ± 9.26	10.80 ± 10.16	29.41 ± 21.21
aCRC (mean)	8.94 ± 9.99	9.44 ± 10.5	9.94 ± 11.1	10.5 ± 11.7	15.2 ± 13.5	30.1 ± 21.1
yCRC (total)	14,895	15,018	15,611	15,987	27,719	75,505
aCRC (total)	286,184	302,074	317,984	336,325	486,911	963,217
Hospitalizations						
yCRC (median)	0	0	0	0	0	3
aCRC (median)	0	0	0	0	0	2
yCRC (mean)	0.127 ± 0.44	0.140 ± 0.49	0.149 ± 0.52	0.150 ± 0.54	0.601 ± 0.88	2.95 ± 1.89
aCRC (mean)	0.235 ± 0.649	0.254 ± 0.686	0.271 ± 0.755	0.292 ± 0.824	0.786 ± 1.10	2.53 ± 1.76
yCRC (total)	325	359	382	384	1543	7577
aCRC (total)	7529	8152	8674	9365	25160	80866
Emergency visits						
yCRC (median)	0	0	0	0	0	0
aCRC (median)	0	0	0	0	0	0
yCRC (mean)	0.00506	0.0222	0.0354	0.0514	0.212	0.484
aCRC (mean)	0.00500	0.0174	0.0366	0.0613	0.164	0.316
yCRC (total)	13	57	91	132	543	1243
aCRC (total)	160	557	1172	1962	5251	10110
Colonoscopies						
yCRC (median)	0	0	0	0	0	0
aCRC (median)	0	0	0	0	0	0
yCRC (mean)	0.00506 ± 0.0812	0.00623 ± 0.0787	0.00429 ± 0.0653	0.00818 ± 0.0943	0.0514 ± 0.228	0.438 ± 0.617
aCRC (mean)	0.00771 ± 0.090	0.00806 ± 0.092	0.00865 ± 0.097	0.00868 ± 0.102	0.0715 ± 0.274	0.372 ± 0.589
yCRC (total)	13	16	11	21	132	1124
aCRC (total)	247	258	277	278	2288	11936

Supplementary Table S2. ICD9 codes for reasons for visits ('complaints')

	ICD9 codes
Potentially related reasons	785, 787, 789, 569, 455, 239, 206, 192, 455
Potentially red flag reasons	02A, 153, 280, 285, 578, 569.3
Specific reasons	
Abdominal pain	02A, 789
Nausea & vomiting	787
Other disorders of intestine	569
Bleeding	241, 578
Hemorrhoid	455
Anemia	280, 285

We categorized symptoms into "potentially related" or "potentially red flag". ICD9 codes were utilized within the MSP database based on the year of outpatient visit. All other ICD9 codes were considered unrelated.

Supplementary Table S3. Top 30 ICD9 codes for yCRC cases in prediagnosis year-1

Rank	ICD9 code	Count	Percent (%) of all ICD9 codes	Diagnosis/Description
1	787	2,029	7.3	Nausea and vomiting
2	780	1,129	4.1	General symptoms
3	789	950	3.4	Other symptoms including abdomen and pelvis
4	02A	914	3.3	Abdominal pain
5	569	742	2.7	Other disorders of intestine
6	781	529	1.9	Symptoms involving nervous and musculoskeletal systems
7	01L	450	1.6	Laboratory abnormality
8	401	439	1.6	Essential hypertension
9	455	438	1.6	Hemorrhoids
10	311	437	1.6	Depressive disorder
11	578	425	1.5	Gastrointestinal hemorrhage
12	153	409	1.5	Malignant neoplasm of colon
13	250	374	1.4	Diabetes mellitus
14	7890	371	1.3	Other symptoms involving abdomen and pelvis
15	280	304	1.1	Iron deficiency anemia
16	782	300	1.1	Symptoms involving skin and other integumentary tissue
17	50B	292	1.1	Anxiety
18	300	289	1.0	Anxiety
19	304	268	1.0	Drug dependence
20	724	251	0.9	Other disorders of the back
21	626	250	0.9	Menstrual disorder
22	785	249	0.9	Abnormal heart beat
23	788	243	0.9	urinary system symptom
24	5693	241	0.9	Rectal bleeding
25	564	239	0.9	functional digestive disorder
26	786	226	0.8	respiratory symptom
27	01Z	213	0.8	anesthetic
28	9	206	0.7	Ill-defined intestinal infection
29	784	199	0.7	Head & neck symptoms
30	555	192	0.7	regional enteritis

Supplementary Table S4. Top 30 ICD9 codes for aCRC cases in prediagnosis year-1

Rank	ICD9 code	Count	Percent (%) of all ICD9 codes	Diagnosis/Description
1	401	26,321	5.4	Hypertension
2	787	24,177	5.0	Gastrointestinal symptoms
3	780	18,299	3.8	General symptoms NYD
4	250	17,389	3.6	Diabetes
5	785	13,702	2.8	Intestinal disorder
6	01L	11,490	2.4	Lab abnormality
7	789	9,210	1.9	Pelvic symptoms
8	781	9,059	1.9	Nervous and MSK symptoms
9	569	9,025	1.9	Other disorders of intestine
10	153	7,412	1.5	Colon cancer
11	02A	7,138	1.5	Abdominal pain
12	578	6,921	1.4	Gastrointestinal hemorrhage
13	366	6,460	1.3	Cataract
14	427	6,344	1.3	Premature beats
15	786	5,935	1.2	Symptoms involving respiratory system and other chest symptoms
16	585	5,743	1.2	Chronic kidney disease
17	280	4,977	1.0	Iron deficiency anemia
18	365	4,873	1.0	Preglaucoma
19	788	4,711	1.0	Symptoms involving urinary system
20	715	4,549	0.9	Osteoarthritis
21	286	4,450	0.9	coagulation abnormality
22	782	4,418	0.9	skin
23	311	4,258	0.9	depression
24	01Z	3,878	0.8	anesthetic
25	428	3,464	0.7	heart failure
26	455	3,397	0.7	hemorrhoids
27	724	3,377	0.7	back pain
28	999	3,276	0.7	iatrogenic complication
29	33A	3,075	0.6	injection
30	285	3,060	0.6	unspecified anemia

Supplementary Table S5. Most frequent presenting complaints* for outpatient visits during prediagnosis year-5 for yCRC and aCRC cases

	yCRC		aCRC		p-value
	**n**	**%**	**n**	**%**	
Unrelated visits	6,066	92.1	135,430	95.8	<0.001
Potential related visits	407	6.2	5,960	4.2	<0.001
Potentially red flag visits	111	1.7	0	0	<0.001
**Specific complaints**					
“Other disorders of intestine” OR “Other symptoms of pelvis”	407	6.2	5,960	4.2	<0.001
Abdominal pain	111	1.7	-		<0.001
**Total visits**	**6,584**		**141,390**		

*Recorded ICD9 codes in the MSP database were used to identify reasons for visits (‘complaints’). All percentages are out of entire subset of presenting complaints for the respective age cohort. These were then mapped as: **1) unrelated visits** for symptoms that may not be connected to a diagnosis of CRC; **2) potentially related visits** for symptoms that may be connected to a diagnosis of CRC (nausea & vomiting, other abdominal or pelvic symptoms; and **3) potentially red flag visits** which include a diagnosis of a malignancy, abdominal pain, gastrointestinal hemorrhage, or iron deficiency anemia, consistent with NICE guidelines. Please refer to **Supplementary Table S2** for ICD9 codes and descriptions.
